# Elevated Plasma Complement C1Q Measured Subacutely after Traumatic Brain Injury Is Associated with Poor Functional Outcome Independent of Initial Injury Severity

**DOI:** 10.1089/neur.2024.0152

**Published:** 2025-02-12

**Authors:** Tracy Butler, Kewei Chen, Abigail Patchell, Xiangling Mao, Dikoma Shungu, Diany Paola Calderon, Jeanne T. Paz, Sudhin A. Shah

**Affiliations:** ^1^ Department of Radiology, Weill Cornell Medicine, New York, New York, USA.; ^2^ College of Health Solutions, Arizona State University, Phoenix, Arizona, USA.; ^3^ Department of Anesthesiology, Weill Cornell Medicine, New York, New York, USA.; ^4^ Gladstone Institute of Neurological Disease, San Francisco, California, USA.; ^5^ Department of Neurology, Kavli Institute for Fundamental Neuroscience, UCSF, San Francisco, California, USA.

**Keywords:** adult brain injury, biomarkers, inflammation, neuroplasticity, recovery, secondary insult, thalamus

## Abstract

Following traumatic brain injury (TBI), secondary processes, including inflammation, contribute significantly to long-term cognitive and functional impairments. Targeting these secondary processes during the subacute period after TBI represents a feasible therapeutic target. This study investigates the role of complement factor 1q (C1Q) in TBI recovery. Motivated by our rodent studies showing that thalamic inflammation post-TBI is dependent on C1Q and that blocking C1Q during the subacute period can prevent thalamic inflammation and improve aspects of TBI outcome, particularly sleep, we measured plasma C1Q levels 3–6 months post-injury in 27 patients with TBI ranging from complicated mild to severe, as well as 30 controls. TBI patients had significantly higher plasma C1Q levels (*p* = 0.031). We assessed the correlation between plasma C1Q and functional outcomes using the Glasgow Outcome Scale-Extended (GOSE), controlling for initial injury severity. Higher plasma C1Q levels were associated with worse functional outcomes (rho = −0.395, *p* = 0.046), independent of initial injury severity. These findings suggest that subacute plasma C1Q may be a novel prognostic biomarker for TBI outcomes. More importantly, subacute plasma C1Q may provide a window into ongoing, C1Q-mediated maladaptive neuroinflammatory processes after TBI that we have shown to be remediable in rodents using a safe-in-human drug that blocks C1Q. Since the initial injury cannot be changed, the ability to intervene subacutely could provide critical therapeutic benefits to the millions affected by TBI each year.

## Introduction

A total of 69 million people worldwide suffer traumatic brain injuries (TBI) each year, and TBI is a leading cause of persistent disability.^1^ Cognitive and behavioral impairments,^2^ post-traumatic epilepsy,^3^ and sleep disruption^4^ are common and debilitating TBI sequelae. Many of these adverse outcomes are thought to be caused by indirect secondary brain injuries that develop over days, weeks, months, or even years after the initial injury, as a consequence of, and at a distance from, the initial impact. While public health and safety initiatives, such as traffic laws, seat belts, and helmets, can prevent or lessen the severity of initial TBI, interventions that affect these secondary processes represent feasible targets to reduce TBI-related disability once an injury has occurred.

Inflammation is a key secondary process following TBI that occurs at the site of injury as well as at remote sites connected via neural pathways. Persistent inflammation in the thalamus—a neural connectivity hub—has been demonstrated after TBI in both rodents^5^ and humans.^6^ Treatments that modulate inflammation have been a focus of TBI therapeutic research for decades, though successful studies in rodents have not translated to humans.^7,8^ These failures can be attributed to many factors, including heterogeneity in human TBI, incomplete understanding of inflammatory and other mechanisms after TBI, and feasibility challenges associated with administering a medication quickly after injury in humans.

We recently showed in rodents that thalamic inflammation after TBI is dependent upon complement factor 1q (C1q) and that administering a medication that blocks C1q at the subacute time point after injury is beneficial, preventing thalamic inflammation, loss of sleep spindles, and development of epileptic spikes nested in sleep spindles.^5^ In humans, there is evidence that plasma C1Q is elevated acutely (within hours) after TBI and associated with greater severity of initial injury and poor outcome.^9^ This suggests that plasma C1Q measured acutely may be a useful biomarker of TBI severity, similar to plasma neurofilament light chain (NfL), which has been linked to TBI severity and outcome in multiple studies.^10^ What is not known, however, is whether C1Q remains elevated at subacute timepoints after TBI and whether subacute C1Q is associated with cognitive/functional recovery from TBI. Our studies in rodents^5^ suggest that understanding the role of C1q at subacute timepoints after TBI may tell us about ongoing, secondary, potentially treatable complement-mediated processes, rather than merely reflecting initial injury severity.

We therefore measured plasma C1Q at the subacute stage (3–6 months after injury) in patients with TBI and assessed the correlation of plasma C1Q with TBI outcome, measured using the Glasgow Outcome Scale-Extended (GOSE).^11^ We measured plasma NfL in a subset of TBI subjects. Based on our findings in rodents that thalamic C1Q at a subacute timepoint after TBI is maladaptive,^5^ we hypothesized that plasma C1Q at an analogous timepoint in humans would similarly be associated with worse outcome. Because in rodents, C1Q in the thalamus corresponds to a delayed, secondary process, remote in time and location from the initial injury, we hypothesized that this association between plasma C1Q and outcome would be independent of the severity of the initial injury (estimated as Glasgow Coma Scale [GCS]).

## Methods and Materials

### Subjects

TBI subjects (*n* = 27) were recruited through rehabilitation and trauma departments. Control subjects (*n* = 30) were recruited through advertisements. All subjects provided informed consent, and all study activities were approved by Weill Cornell’s Institutional Review Board. TBI subjects had sustained a complicated mild (GCS 13–15 with intracranial lesion) or moderate-to-severe TBI (GCS ≤12). Control subjects had no history of TBI and were free from medical and psychiatric illness and substance abuse. TBI subjects underwent blood draw and were administered the GOSE^11^ to assess post-TBI function 3–6 months after injury as part of a larger assessment battery.

### Blood assays

Plasma C1Q was measured at a commercial clinical laboratory (Arup) using radial immunodiffusion assay. In a subset of TBI subjects (*n* = 23), plasma NfL levels measured at the Clinical Neurochemistry Laboratory, University of Gothenburg, Sweden, using a single-molecule array were available. NfL results in these subjects have been reported previously.^12^

### Statistical analyses

Analyses were performed in SPSS for Windows (version 29.0, Chicago, IL).

*t*-Test and chi-square were used to assess demographic and C1Q differences between TBI subjects and controls.

Within TBI subjects, bivariate correlations between variables of interest were assessed using Spearman’s test. Variables of interest were plasma C1Q, plasma NfL, GCS (reflecting initial injury severity), GOSE (reflecting functional outcome), and days between injury and blood sampling.

Partial Spearman correlation was performed to determine the association of C1Q and NfL with outcome (GOSE) while controlling for initial injury severity (GCS). This analysis was also performed when controlling for both GCS and days between injury and blood sampling.

## Results

Participant characteristics are shown in Table 1. Control and TBI subjects did not differ by age or sex.

**Table 1.** Participant **Characteristics and C1Q and NfL Plasma Levels in TBI versus Control Subjects.** Results Are Presented as Mean ± SD (Range). TBI Subjects Had Higher Plasma C1Q Levels as Compared with Controls (*t* = −2.208, df = 55, *p* [2-Sided] = 0.031)

	Controls (30 subjects)	TBI (*n* = 27 subjects)	*p* value
Sex	16 females, 14 males	10 females, 17 males	0.33
Age (years)	44.2 ± 13.1 (23–65)	45.0 ± 18.9 (19–82)	0.85
C1Q (µg/ml)	130.7 ± 37.7 (59–208)	152.07 ± 35.07 (91–208)	**0.031**
Glasgow Coma Scale (GCS)	N/A	11.7 ± 3.96 (3–15)	N/A
Glasgow Outcome Scale-Extended (GOSE)	N/A	5.67 ± 1.61 (3–8)	N/A
Days between injury and blood sampling	N/A	149.48 ± 41.4 (94–241)	N/A
NfL^a^ (pg/mL)	N/A	90.59 ± 115.18 (7.0–507)	N/A

C1Q, complement factor 1Q; NfL, neurofilament light chain.

^a^ NfL was only available in 23/27 TBI subjects.

TBI subjects had higher plasma C1Q levels as compared with controls (*t* = −2.208, df = 55, *p* [2-sided] = 0.031).

Among the variables of interest (C1Q, NfL, GCS, GOSE, days since injury), the only significant correlations were between NfL and GCS (rho = −0.525, *p* = 0.01, *n* = 23) and between NfL and days since injury (rho = −0.586, *p* = 0.003, *n* = 23).

**FIG. 1.**
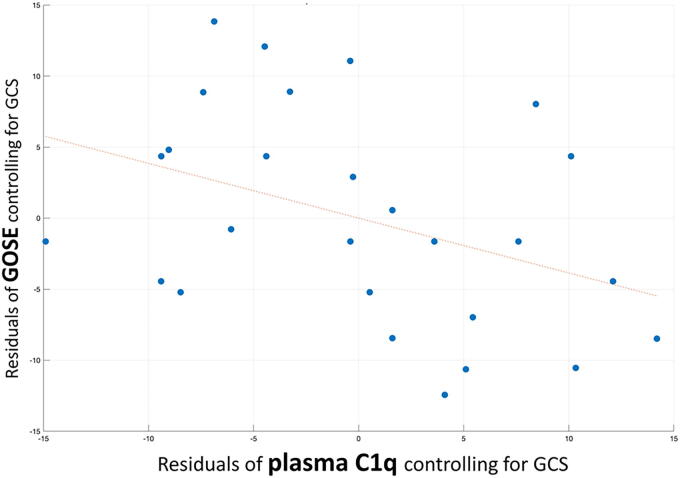
Higher plasma C1Q was associated with worse functional outcomes in 27 subjects with TBI, and this association was independent of initial injury severity. Partial regression plot showing significant partial correlation (rho = −0.395, df = 24, *p* = 0.046) between plasma C1Q and functional outcome from TBI (GOSE score) when adjusting for initial injury severity (GCS). C1Q, complement factor 1Q; GOSE, Glasgow Outcome Scale-Extended; GCS, Glasgow Coma Scale.

When controlling for initial injury severity (GCS), plasma C1Q was significantly associated with GOSE^11^ (rho =−0.395, df = 24, *p* = 0.046) such that higher plasma C1Q was associated with lower GOSE score, reflecting worse functional outcome from TBI. Results were similar when adjusting for both GCS and days between injury and blood draw (rho = −0.375, df = 23, *p* = 0.064). A partial regression plot showing the correlation between C1Q and TBI outcome when adjusting for GCS is shown in Figure 1.

NfL was not associated with functional outcome (GOSE score) when adjusting for initial injury severity (GCS) or both initial injury severity and days since injury (*p* > 0.1).

## Discussion

We found that plasma C1Q measured subacutely (∼5 months; 94–241 days) after injury in 27 subjects with TBI ranging from complicated-mild to severe was significantly elevated as compared to 38 controls. This finding is in accord with one prior study measuring C1Q acutely (within hours) after TBI,^9^ and contributes to mounting evidence that the complement system is dysregulated after TBI in humans, in accord with animal studies.^5,13^

We found that plasma NfL measured subacutely was significantly associated with initial injury severity estimated as GCS. NfL also correlated inversely with time since injury. These NfL findings are expected and in accord with multiple studies showing that NfL—an axonal protein released from brain to blood with injury—reflects severity of brain tissue disruption at the time of injury.^10^

We did not detect a significant correlation between subacute C1Q and initial injury severity. This finding at the subacute stage contrasts with results from a prior study, which showed that C1Q measured hours after injury correlated closely with initial GCS.^9^ While this discrepancy could relate to our small sample size, we believe it is more likely due to the different processes occurring at acute versus subacute time points. Rather than being a marker of initial injury severity, such as NfL, plasma C1Q measured subacutely after human TBI may be a window into the delayed, C1Q-mediated maladaptive process of thalamic inflammation we demonstrated in a rodent TBI model.^5^

The most important and novel finding of this study is that plasma C1Q measured ∼5 months after injury was significantly associated with TBI recovery and functional outcome, and this association was independent of initial injury severity. These results suggest that plasma C1Q measured subacutely (i.e., months) after injury may be a novel prognostic indicator for TBI outcome. But, critically, plasma C1Q may be more than a TBI prognostic biomarker. It may provide mechanistic insight in humans into a maladaptive, C1Q-mediated thalamic neuroinflammatory process that occurs after the initial injury, and which we have shown in rodents to be remediable using an existing anti-C1Q medication.^5^

### Limitations

The sample size for this study is quite low, and we do not have acute measures of C1Q, which would be needed to fully understand the time course of C1Q changes after TBI. Low sample size also makes assessment of potential sex differences challenging. We do not have measures of other complement proteins. We do not have measures of peripheral injury, which might be expected to affect C1Q and other complement components.

## Conclusion

Plasma C1Q measured subacutely after TBI is associated with TBI outcome independent of initial injury severity, differing importantly from other TBI biomarkers such as NfL. Rather than reflecting the severity of the original injury—which cannot be changed—subacute C1Q may reflect maladaptive complement activation as a secondary/indirect mechanism of injury. Given the availability of safe-in-human medications that block complement components, including C1Q,^14^ and preclinical evidence that blocking C1Q improves post-TBI outcome by reducing thalamic inflammation, normalizing sleep, and preventing epileptiform discharges,^5^ understanding the role of complement-mediated processes after TBI could have near-term therapeutic implications.

## Transparency, Rigor, and Reproducibility Summary

This is a pilot analysis measuring complement C1Q in plasma samples available in a subset of subjects enrolled in a larger TBI study (R01NS102646). The analysis plan was not formally pre-registered, and the sample size was not pre-specified. The analysis is based on cross-sectional data from all subjects who provided a blood sample. De-identified data from this study will be made available upon request of the 1st or last author and institutional approval of a data sharing-agreement.

## Abbreviations Used

TBI: traumatic brain injury
GOSE: Glasgow Outcome Scale-Extended
NfL: neurofilament light chain
